# P01.19. Oxytocin Receptor Gene (OXTR) variation is associated with enhanced affective and placebo conditioning to touch-based complementary interventions

**DOI:** 10.1186/1472-6882-12-S1-P19

**Published:** 2012-06-12

**Authors:** S Jain, K Aschbacher, N Bat, P Mills, W Jonas, J Ives, C Carter, J Connelly

**Affiliations:** 1 Samueli Institute / University of California San Diego, San Diego, USA; 2 Samueli Institute / University of California San Francisco, San Francisco, USA; 3 Samueli Institute, Alexandria, USA; 4 University of California San Diego, San Diego, USA; 5 University of Illinois-Chicago, Chicago, USA; 6 University of Virginia, Charlottesville, USA

## Purpose

Patients often report reductions in negative affect in response to biofield and touch interventions. While reasons for improvement remain unclear, current theory and evidence suggest the need to examine oxytocin as well as placebo mechanisms. We investigated changes in negative affect (NA) during an acute session of verum or touch-based healing, and related NA changes to placebo elements, plasma oxytocin changes, and the oxytocin receptor (OXTR SNP rs53576) polymorphism (which has been associated with psychological sensitivity to social context).

## Methods

Thirty-two fatigued breast cancer survivors received 60-minute touch-based verum or mock-healing intervention. After receiving the first session, patients immediately rated their beliefs on treatment helpfulness in terms of fatigue, well-being, and immune function. Within a week, patients received a second session, during which pre-post session NA (Profile of Mood States) and blood draws were obtained and assayed for plasma oxytocin levels (ELISA) and OXTR genotyping. Data were analyzed using Pearson correlations and general linear models.

## Results

Both verum and sham groups showed significant pre-post session decreases in NA (p=.005) with no group differences (p=.31). Pre-post session, NA decreases were marginally associated with increases in plasma oxytocin (r = -.35, p=.06). Pre-session NA and plasma oxytocin did not differ by OXTR genotype (p>.33). However, A-allele carriers showed significantly greater reductions in pre-post NA than G/G carriers (t(30)=2.702, p=.011, n=19). A-allele carriers also uniquely showed significant associations with belief and NA decrease, such that for A-allele carriers only, prior-rated belief in treatment helpfulness was associated with greater decreases in negative affect (r=-.473, p=.04, n=19).

## Conclusion

OXTR rs53576 A-allele carriers show greater reductions in NA and unique associations with belief and NA reductions in response to touch-based interventions. Results suggest that psychological and placebo conditioning responses to touch-based interventions may be predicted by biological sensitivity to social context.

